# Applicability and usage of dose mapping/accumulation in radiotherapy

**DOI:** 10.1016/j.radonc.2023.109527

**Published:** 2023-02-10

**Authors:** Martina Murr, Kristy K. Brock, Marco Fusella, Nicholas Hardcastle, Mohammad Hussein, Michael G Jameson, Isak Wahlstedt, Johnson Yuen, Jamie R McClelland, Eliana Vasquez Osorio

**Affiliations:** a Section for Biomedical Physics, Department of Radiation Oncology, University of Tübingen, Germany; b Department of Imaging Physics and Department of Radiation Physics, The University of Texas MD Anderson Cancer Center, USA; c Department of Radiation Oncology, Abano Terme Hospital, Italy; d Physical Sciences, Peter MacCallum Cancer Centre & Sir Peter MacCallum Department of Oncology, University of Melbourne, Australia; e Metrology for Medical Physics Centre, National Physical Laboratory, Teddington, United Kingdom; f GenesisCare New South Wales, School of Clinical Medicine, Faculty of Medicine and Health, University of New South Wales, Australia; g Department of Health Technology, Technical University of Denmark, Anker Engelunds Vej 1, Bygning 101A, 2800 Kongens Lyngby; h Department of Oncology, Centre for Cancer and Organ Diseases, Copenhagen University Hospital - Rigshospitalet (RH), Blegdamsvej 9, 2100 Copenhagen; i Department of Oncology, Copenhagen University Hospital - Herlev and Gentofte (HGH), Borgmester Ib Juuls Vej 7, 2730 Herlev, Denmark; j St George Hospital Cancer Care Centre, Kogarah, NSW 2217; k South Western Clinical School, University of New South Wales, Sydney; l Ingham Institute for Applied Medical Research, Sydney, NSW, Australia; m Centre for Medical Image Computing and Wellcome/EPSRC Centre for Interventional and Surgical Sciences, Dept of Medical Physics and Biomedical Engineering, UCL, United Kingdom; n Division of Cancer Sciences, Faculty of Biology, Medicine and Health, The University of Manchester, M20 4BX Manchester, United Kingdom

**Keywords:** Dose mapping/accumulation, Deformable image registration (DIR), DIR uncertainties, Impact of dose mapping uncertainties, Anatomical changes, Dose mapping/accumulation landscape (DMAL)

## Abstract

Dose mapping/accumulation (DMA) is a topic in radiotherapy (RT) for years, but has not yet found its widespread way into clinical RT routine. During the ESTRO Physics workshop 2021 on “commissioning and quality assurance of deformable image registration (DIR) for current and future RT applications”, we built a working group on DMA from which we present the results of our discussions in this article. Our aim in this manuscript is to shed light on the current situation of DMA in RT and to highlight the issues that hinder consciously integrating it into clinical RT routine.

As a first outcome of our discussions, we present a scheme where representative RT use cases are positioned, considering expected anatomical variations and the impact of dose mapping uncertainties on patient safety, which we have named the DMA landscape (DMAL). This tool is useful for future reference when DMA applications get closer to clinical day-to-day use.

Secondly, we discussed current challenges, lightly touching on first-order effects (related to the impact of DIR uncertainties in dose mapping), and focusing in detail on second-order effects often dismissed in the current literature (as resampling and interpolation, quality assurance considerations, and radiobiological issues).

Finally, we developed recommendations, and guidelines for vendors and users. Our main point include: Strive for context-driven DIR (by considering their impact on clinical decisions/judgements) rather than perfect DIR; be conscious of the limitations of the implemented DIR algorithm; and consider when dose mapping (with properly quantified uncertainties) is a better alternative than no mapping.

Radiotherapy (RT) aims at treating cancer by delivering radiation to the affected zones (target). In RT planning, a dose distribution is generated on a medical image. Dose distributions are often highly heterogeneous and conformal with steep dose gradients in anatomy close to the target which may move, deform, and/or respond to treatment. It is useful to transfer the dose distribution from one image to another to compare and/or accumulate doses to specific anatomical sub-volumes. Transferring or mapping dose distributions requires aligning the underlying images, assuming any transformation describing the geometric mapping between the two frames of references of the images, can be applied to their corresponding dose distributions [1] In Fig. 1a–b, we present a scheme detailing this assumption. Note we refer to the source image as the image associated with the dose to be mapped. Conversely, the destination image defines the grid where we want to map the dose.

Aligning images is done using image registration, which aims at finding the transformation that optimally aligns corresponding anatomy between two images. Rigid transformations, consisting of rotations and translations, are often used to correct for global misalignments (correcting patient positioning). However, rigid transformations are not sufficient to resolve the misalignment due to local changes [2], or large position changes in patient set-up [3,4]. In these cases, deformable image registration (DIR), is preferred [5–7]. The inputs for DIR include two images, a fixed and a floating image, and the result is a non-rigid transformation. The transformation (T in Fig. 1) is often represented as a deformation vector field (DVF). DVFs are composed by a set of vectors originating from the voxel positions in the fixed image and pointing to the corresponding locations in the floating image (typically not coinciding with the centre of a voxel). Specifically, T maps point coordinates from the fixed grid to the floating frame of reference.

Mapping the source dose distribution to the destination grid can be achieved by either gridded resampling (which pulls or “fishes out” the dose from the floating image), or scattered resampling (which pushes or shoots the dose, as in archery, to the floating image). Depending on the selected strategy, the source/destination image takes the role of the fixed or floating image in the registration, Fig. 1c. For several applications, the mapped dose is then summed up with another dose, which is known as dose accumulation.

Uncertainties in the registration will introduce dose mapping uncertainties. An important factor affecting registrations is the degree of anatomical variation. Small differences may be accurately registered by most algorithms while complex changes may not. However, a limited number of algorithms attempt to address complex changes [2,3], such as sliding tissue [4,5], (dis)appearance of tissue (including different tumour regression modes [6] and surgical interventions [7–11]). Often these advanced algorithms are only available for research. Other factors can also affect the quality of the registration, such as image acquisition artefacts, lack of contrast, choice of parameters, etc. [1,12]. These complex changes and other factors lead to large uncertainties in the registration, and hence uncertainties in the mapped dose, particularly in regions where high dose gradients are present (Fig. 2). Therefore, quantifying dose mapping uncertainties would need to account for both registration uncertainties and dose characteristics.

Dose mapping can be complex and challenging. It is often not clear what the ‘correct’ transformation is, and exactly how this should be used to resample and accumulate the doses. Uncertainties in dose mapping could impact treatment outcomes. An extreme example is if new fractions are re-optimised accounting for estimated delivered dose (mapped with significant errors), which can ultimately result in relapse (if the target was seriously underdosed) or severe side effects (if normal tissues are overdosed). Consequently, determining the proper registration needs to consider at least these two aspects: degree of anatomical variations and the impact of dose mapping uncertainties.

In this manuscript, we will present considerations demonstrating that DMA is more than just applying the results of a registration, as correct dose mapping is not guaranteed even with “perfect” registrations. We also propose a visualisation aid to exemplify the variation in anatomy and impact of uncertainties on the patient’s treatment for a range of use cases. We finish the paper with a list of recommendations aimed at users and vendors. Notice that we will not discuss image registration in depth, as several reviews are available in the literature [1,3,13,14], including reviews focussed on DMA [2,15].

## Workshop discussions

Discussions were held during the ESTRO Physics workshop 2021 on “commissioning and quality assurance of DIR for current and future RT applications”. This workshop included two focussed online sessions with more than 20 participants. During the second session aspects identified in the first session were discussed in groups. The result of the discussions in the “DMA” group forms the basis of this article.

## Dose mapping/accumulation landscape (DMAL)

A direct outcome of these discussions was a scheme representing the current landscape of DMA use cases. The scheme was based on two axes: the degree of anatomical variations expected, and the impact of dose mapping uncertainties on patient safety.

### Anatomical variations

The degree of anatomical variations between the images may challenge the registration algorithms [16]. Anatomical variations can be divided into two main categories in RT applications, intraand inter-patient (Fig. 3a).

For intra-patient applications, the degree of anatomical variations often increases with the time difference between images: in an extreme example, large anatomical variations are observed between medical scans over lifetime (newborn vs adult). This is particularly relevant in the re-irradiation context, where dramatic changes can be caused by treatment and time. Alternatively, smaller changes can be expected when registering images taken minutes apart, such as intra-fraction images [17].

For variations during treatment, we distinguish between inter-[18] and intra-fraction changes [19,20]. These include disappearance and appearance of tissue and content (e.g. bladder/rectum filling [21,22]), and tissue changes (e.g. tumour regression/growth, weight loss) [23–25]. The changes vary from small (millimetres) to large (centimetres) [24,26,27] (Fig. 3a). The range varies depending on several factors like tumour site, and healthy surrounding tissues [28,29]. Furthermore, anatomical variations occur in all directions often leading to complex deformations [30–32]. These complex changes inherently challenge the assumption of 1-to-1 anatomical mapping made by most DIR algorithms.

For inter-patient dose mapping applications, anatomical variations have a different meaning, representing anatomical differences between individuals rather than changes over time. Interpatient dose mapping is used to explore local association of doses and outcomes [33], or assess biological effects [34]. The magnitude of inter-patient differences tends to be larger than most intrapatient changes. Moreover, “corresponding” anatomy is not well defined, as there is not a strict 1-to-1 anatomical mapping due to the natural variability between patients.

### Impact of uncertainties

The impact of uncertainties depends on the intended use of the mapped dose, with increasing impact to applications close to a particular patient’s pathway, Fig. 3b. For this axis, we propose three main levels with no strict borders. With low impact for patient’s safety, we considered the use cases where mapped doses are used to quantify, like delivered doses in retrospective studies [35], or dose variations across large groups [36,37]. With an intermediate impact, we considered applications where the mapped/accumulated dose is used to inform by estimating global statistics. For example, when dose from an initial treatment is mapped to a subsequent image for re-treatment, and only DVH statistics are used to inform dose constraints in a re-irradiation plan [38]. Last, with the highest impact for patient’s safety, we consider cases when the (voxel-by-voxel) mapped doses are incorporated to change a patient’s treatment. For instance, online adaptive RT (ART), when a fraction is adapted on the spot using an estimation of the delivered dose. Uncertainties in the mapped dose due to inelastic tumour regression (visible tumour regression with the healthy surrounding tissue staying in place [25]), may result in underdosing invisible disease which could increase the risk of local recurrence. These are some of the reasons for cautious clinical integration of this tool.

### Current landscape

The dose mapping/accumulation landscape (DMAL), Fig. 4, is populated with relevant example use cases that highlight how these dimensions vary for different use cases. A selection of three cases are discussed in Supplement 1.

## Identification of current challenges

Nine participants from the original workshop met five times between January and April 2022. During these meetings, we discussed articles highlighting critical issues and current challenges in implementation and clinical adoption of dose mapping, Supplement 2. Due to space constraints, we present our considerations for four issues and challenges in detail.

We hypothesise that there are first- and second-order effects on the uncertainties of DMA. The first-order effects relate to the impact of registration uncertainties on dose mapping. Mentioned earlier, it is often impossible for the registration result to ‘accurately’ map the anatomy between two images. We argue that considering what is an appropriate registration and the impact of uncertainties for a particular application is more relevant than discussing its absolute ‘accuracy’, as we lack a ‘ground truth’ mapping with which to validate the accuracy. Second-order effects, including issues such as energy/mass transfer vs dose mapping, resampling/interpolation, etc., also contribute to dose mapping uncertainties, but with a smaller impact. For dose accumulation, biological uncertainties such as using a given ɑ/β value for EQD2 or BED, or the validity of the LQ model, are relevant.

Moreover, uncertainties could impact the applications in a systematic or random manner. For example, when a prior treatment’s dose distribution is transferred onto a subsequent re-irradiation scan any mapping uncertainty will have a systematic impact on the second treatment. Conversely, uncertainties associated with accumulating the dose of a fractionated treatment can typically be considered random.

### First-order effects

Evaluating the correctness of a registration is extremely challenging as ground truth is commonly unavailable. Phantoms or biomechanical models are proposed to quantify registration errors at commissioning [1,39]. For patient-specific applications, tools still need to be developed and used in daily practice [40]. Alternatively, methods to quantify dose mapping uncertainty as maps [41,42] are available, section Quality Assurance. However, dose uncertainty estimations only assess consistency. Determining whether the registration is correct goes beyond quantifying consistency. Attempts to study this aspect with deformable phantoms have been reported [43]. Auxiliary structures/landmarks can also be used to determine the degree of accuracy, as suggested in TG-132 [1] or dense landmark clouds, as suggested by Paganelli [40]. However, the validity of these metrics is limited to the region where these contours/points are defined. Therefore, this is still an open issue which requires careful consideration when applying DIR in daily clinical applications.

As shown in Fig. 2, the registration uncertainties interplay with the dose distribution characteristics, particularly dose gradients [33,44]. The theoretical impact of registration uncertainties to map a dose distribution can be quantified using the distance to dose difference (DTD) [44]. DTD indicates the local admissible registration uncertainty that would keep dose mapping uncertainty below a given tolerance. They found that registration uncertainties of maximum 1 mm would be allowed in regions of steep dose gradients, while uncertainties of > 20 mm are acceptable in regions of low dose gradients, for an IMRT lung dose distribution using a 5 % of the prescribed dose as tolerance.

Generally, DIR is performed for mono- (such as CT-CT) or multimodal (CT-CBCT or CT-MR, etc) images. In multimodal DIR, the differences in the images pose an additional challenge. For example, due to CBCT noise, artefacts, reduced image quality and limited field of view/length, impairs CT-CBCT DIR [45,46]. Challenges in MR-CT DIR occur e. g. by MRI of the lungs which show less structure than CT [47] or by CT images of the prostate which do not show clear boundaries of the organ [48].

Mentioned earlier, there is often no true 1-to-1 mapping between images, especially for (dis)appearance of tissue or anatomies from different individuals. However, the vast majority of registration algorithms attempt to obtain a 1-to-1 mapping between the images (especially diffeomorphic registration algorithms) [49–51]. Even though this may not be a good representation of the true mapping between the images, it is recommended to use transformations that represent a 1-to-1 mapping when resampling dose (positive Jacobian determinant).

Finally, RT accounts for estimates of accuracy at each stage, including dose distributions calculated on grids of 1–3 mm and machines that are accurate to within 2 % or 2 mm. A first goal should be to aim for DIR uncertainties that align with the estimates of the other steps, so that DIR-based DMA is not an outlier in the overall process [52].

### Second-order effects

#### Resampling and interpolation

There has been some discussion in the literature on how dose distributions (represented as images) should be resampled when deforming them with a transformation. Two distinct approaches have been proposed: ‘direct dose mapping’ (DDM) and ‘energy/-mass transfer’ (EMT) [2,53]. DDM directly resamples the dose, whereas EMT first converts the dose into energy and mass, resamples the energy and mass separately, and then calculates the dose from the resampled energy and mass. Some publications claimed or implied that the EMT method is based on more sound physical principles than the DDM method, and therefore gives more correct results [2,53]. We argue that this is based on a misconception that voxels in an image represent discrete anatomical units rather than a discrete sampling of an underlying continuous function. Better understanding and rigorous application of sampling theory would help demonstrate that if the dose, energy, and mass images are resampled correctly, then the same results should be achieved regardless of whether the dose is first converted into energy and mass or not. Therefore, the differences between DDM and EMT comes down to how the resampling is implemented.

Differences between results of the DDM and EMT methods presented in the literature are due to differences in the way the dose images and energy/mass images are interpolated, with neither of the methods following what is commonly considered as best practice for resampling images. We will now highlight some of the main sources of confusion and issues that should be considered when resampling a dose/energy/mass image using a 1D example, Fig. 5. Then, we will provide recommendations on how to best resample dose distributions. For a more detailed discussion of these issues and others related to image resampling see [54–57].

**Fig. 1.**
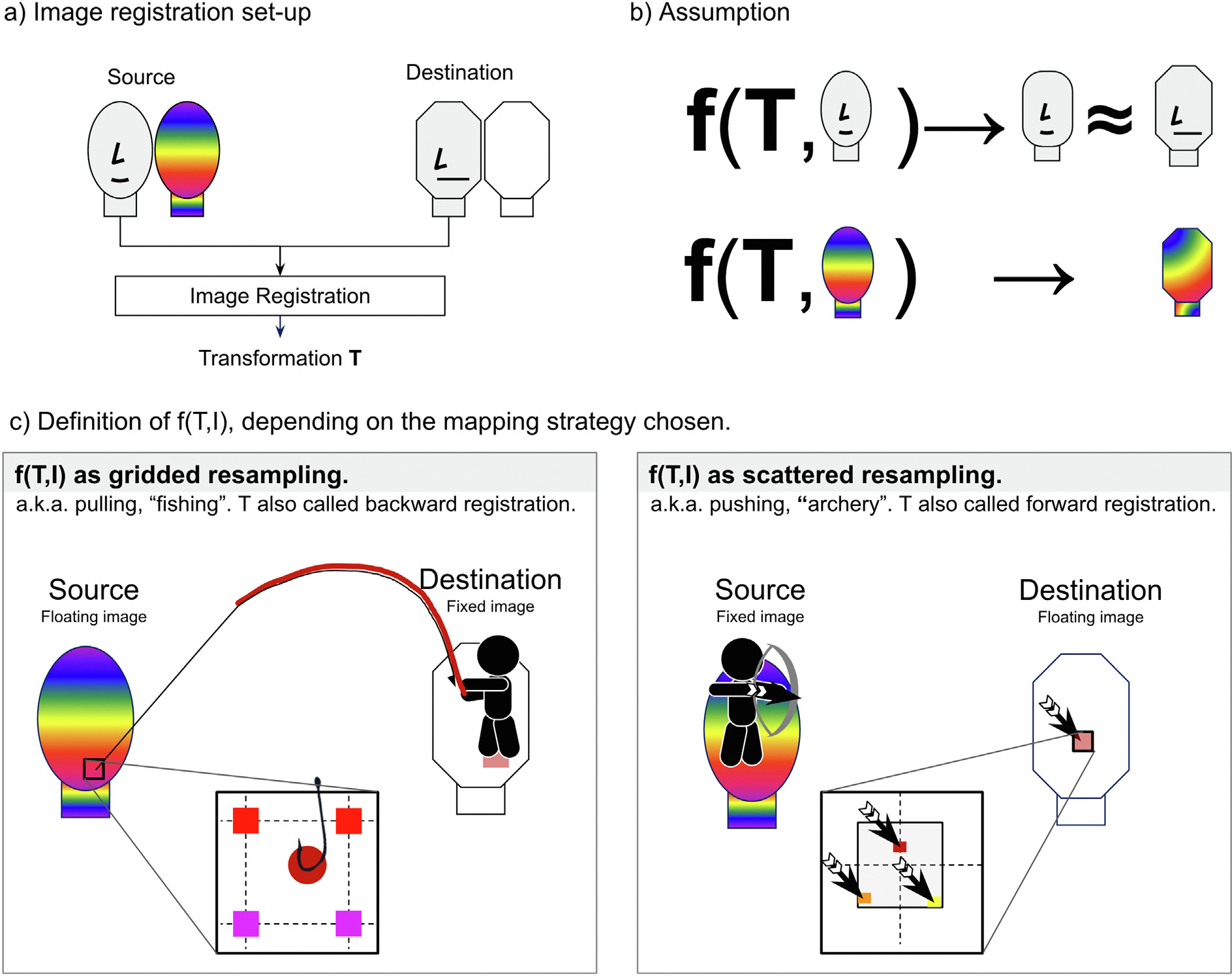
Schematic presenting the use of image registration to map dose distributions. a) Registration set-up. Registration is performed between the source and destination images, here presented in black and white drawings, aiming at mapping the source dose distribution to the destination grid. b) Dose mapping using transformation T, showing the underlying assumption that the registration aligning the pair of images is valid to map any spatially correlated image, such as dose distributions. c) Application of the transformation, depending on the used mapping strategy. Note we distinguish between source/destination and fixed/floating images. Source image is the image that is associated with the dose to be mapped. Destination image is where we want to map the dose. Fixed and floating images are the roles these images take in the registration process.

**Fig. 2.**
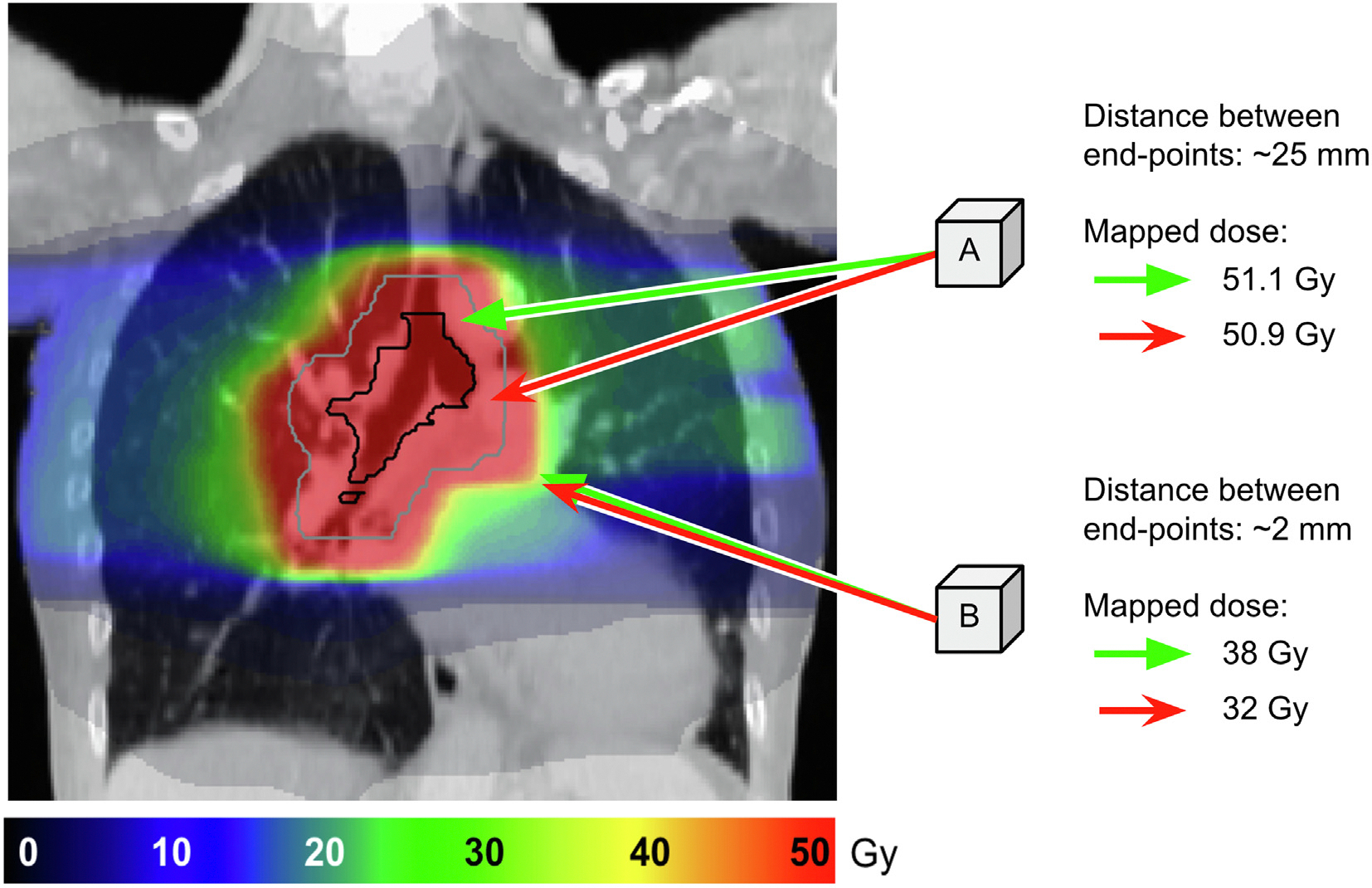
An illustrative case demonstrating the effect of registration uncertainties on dose mapping, and its interplay with dose gradients. For each voxel, A and B, two arrows are shown: 1) a red arrow representing an “erroneous” vector resulting after image registration, 2) a green arrow representing the “correct” vector. Even though there is a large distance between the end-points of two arrows for voxel A, its mapped dose differs slightly. On the other hand, the distance between the end-points for voxel B is small (below “accepted thresholds”), but the mapped dose differs considerably.

**Fig. 3.**
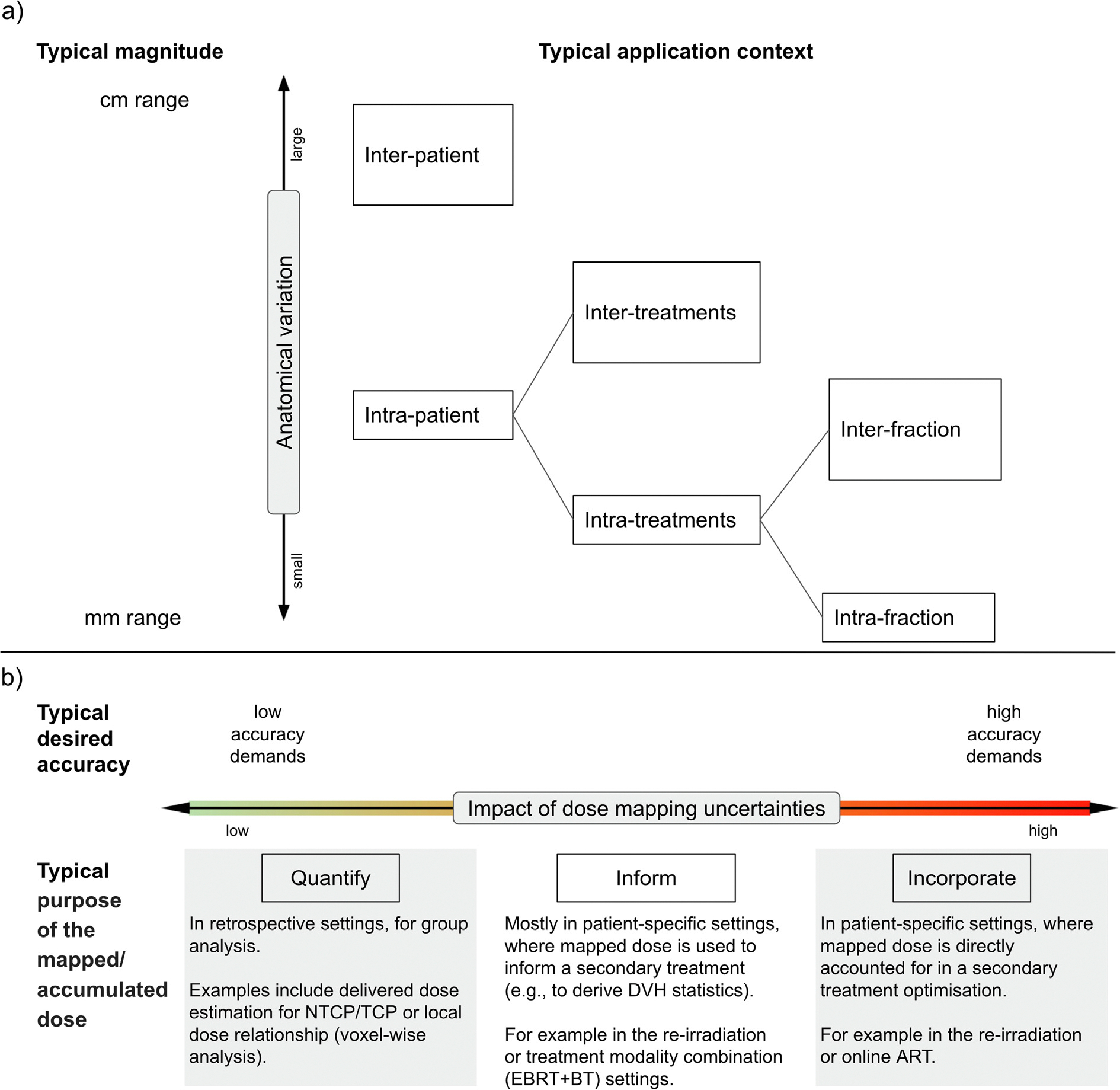
Illustration of the DMAL axes. a) Expected anatomical variations. b) Impact of dose mapping uncertainties on patient safety.

One essential difference between the DDM and EMT methods, as presented in the literature, is the use of ‘pull’ vs ‘push’ resampling or interpolation, Fig. 1c. DDM uses pull interpolation whereas EMT uses push interpolation. In general, pull interpolation is preferred when resampling images as the interpolation step occurs on a regular grid, whereas push interpolation requires interpolating scattered data points. Depending on the method used, scattered data interpolation can lead to undulating artefacts and even ‘holes’ in the resampled image (shown in the EMT method [53] exemplified in Fig. 5c) or relies on methods that are considerably more computationally demanding. This is the reason that all registration algorithms use pull interpolation in their internal optimisation (to the best of our knowledge).

Push interpolation has been used for EMT to ensure that the overall energy/mass was conserved during resampling. Notice that mass can also be preserved when using pull interpolation by multiplying the resampled image by the local volume change (Jacobian determinant), as proposed for mass-preserving registration algorithms [58,59]. Note, such mass-preserving registration algorithms should not be used for most dose resampling applications, as the assumption of mass preservation between the images is not valid (for example due to (dis)appearing tissue or different patients). Our recommendation is to always use pull interpolation when resampling either dose, energy or mass while accounting for local volume changes.

Another cause of the differences between DDM and EMT results in the literature is that the mappings are required in the opposite directions, so separate registrations are performed for each method (swapping the fixed and floating images, Fig. 1c). Inconsistencies between the registration results used for each method (unless an inverse consistent registration is used, which was not the case in [53]), further contribute to the differences in the results observed between the two methods.

On a more technical side, the choice of interpolation method or kernel is key when resampling an image. Linear interpolation is the most well-known and widely used interpolation method. It is very fast but it introduces a small amount of blurring to the resampled image. Other interpolation methods, such as cubic convolution, windowed-sinc (with Lanczos, Blackman or Welch kernels) or spline-based methods, introduce less blurring but can lead to ringing, where the maximum/minimum values in the resampled images are larger/smaller than the maximum/minimum values in the original image [56,57,60], potentially introducing negative dose/energy/mass values. The blurring introduced from a single application of linear interpolation is small and unlikely to have a large impact, however, if the image is resampled multiple times the blurring will accumulate and cause noticeable degradation of the resampled images. This effect will have impacted the evaluation of the DDM method presented in [53] where the dose was resampled 3 times (first resampled onto the source CT image grid, then onto the target CT image grid, and finally resampled onto the target dose image grid) introducing unnecessary blurring to the result. Therefore, we strongly recommend avoiding resampling an image multiple times, instead composing the transformations and only resampling with the final composed transformation.

The final issue we want to highlight is aliasing. This occurs when the original image contains higher frequency information than can be represented in the resampled image, either because the resampled image has a lower resolution or because the transformation causes parts of the image to be compressed. When aliasing occurs the high frequency information incorrectly appears as lower frequencies, which in practice can lead to structures arbitrarily appearing brighter or darker than they should in the resampled image [56,57]. Therefore, we would recommend using a spatially varying interpolation kernel, as described in [55] when the transformation contains regions with large compressions (by a factor of 2 or more) within regions of a high dose gradient.

#### Quality assurance considerations

Quality assurance (QA), defined as the procedures and processes followed to ensure that the quality of each dose mapping is maintained, is essential in clinical practice. However, this is difficult to implement due to unknown ground truth and uncertainties that arise from patient-specific characteristics (for example, cervix-uterus inter-fractional changes [61]). In addition, dose accumulation QA consists of several essential steps: ensuring the appropriate mapping/accumulation workflow was followed, QA of DIR, QA of dose mapping, and finally a review of all steps [1].

**Fig. 4.**
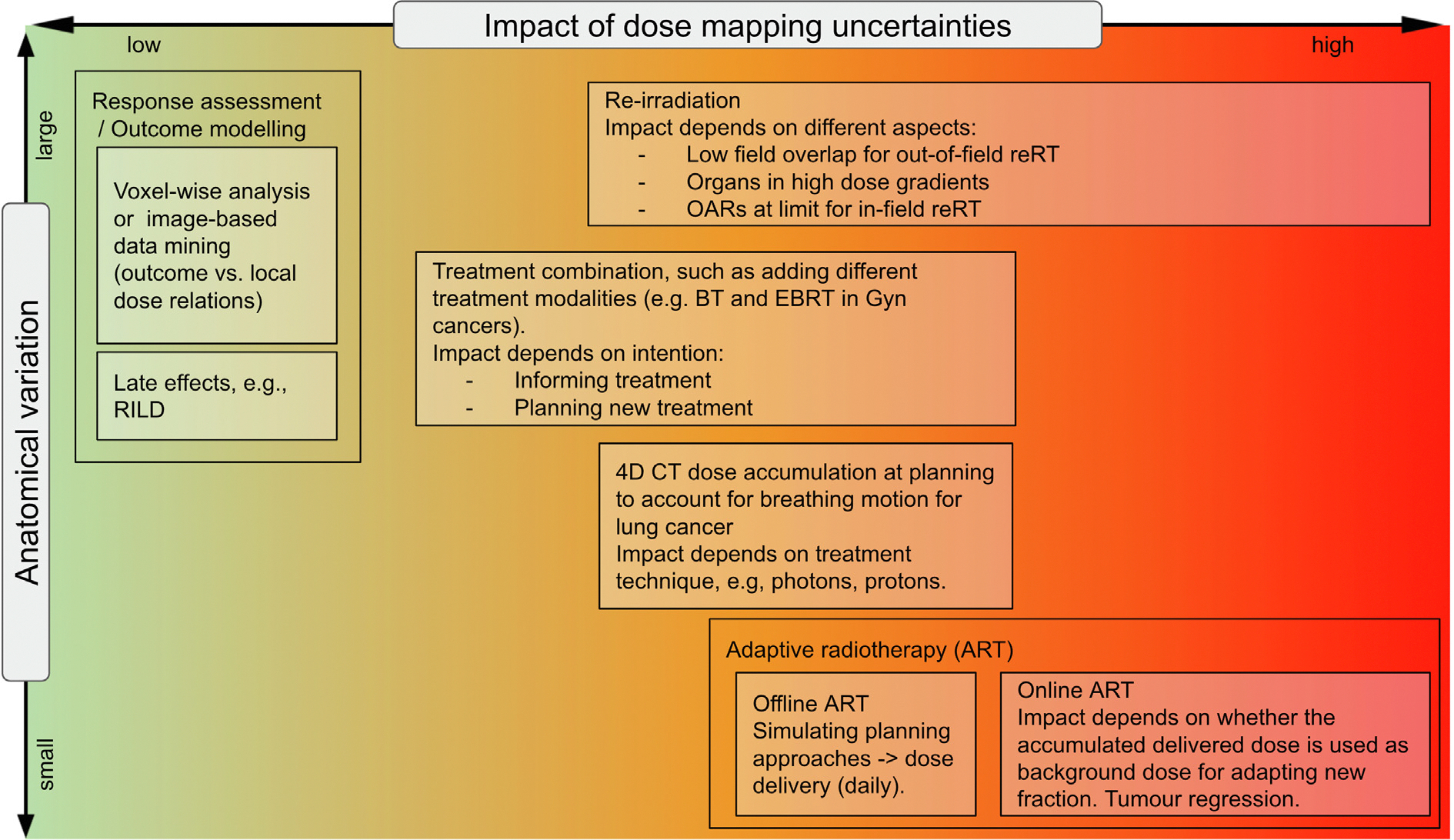
DMAL, presenting the current landscape of use cases. The span of each box represents the typical ranges in the anatomical variation and expected impact of dose mapping uncertainties for a given use case.

**Fig. 5.**
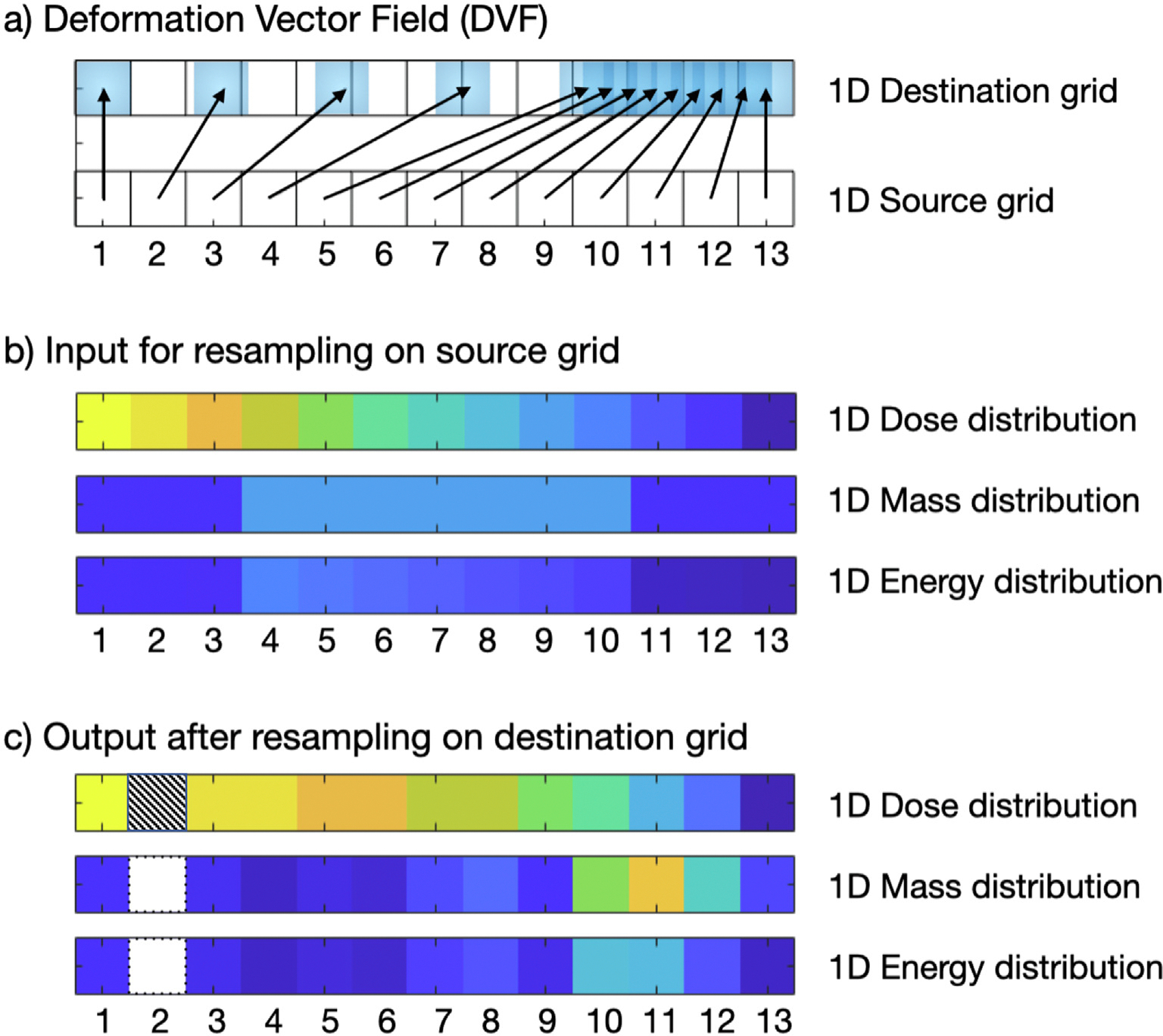
1D scheme highlighting the main problems of using ‘energy/mass transfer’ resulting from naive use of push interpolation. a) The DVF used to push the data representing a constant expansion over 1/3 of the image and a constant compression over the other 2/3, with the 2nd pixel having no mapped pixels. b) The input data. c) The resampled data using the EMT method described in [53]. For each voxel in the dose distributions shown in b) and c), the value corresponds to energy divided by mass. Note the 2nd pixel of the resampled energy and mass distributions corresponds to a ‘hole’ (value of 0), which results in an undefined mapped dose value (hatched pixel). Additionally, the energy and mass distributions contain undulations that would not be expected from the DVF (as it represents constant expansion/compression) and the dose has an undesirable ‘step-like’ appearance (pixel 3 vs 4 having the same value, same for 5 vs 6 and 7 vs 8).

Determining the best workflow for DMA is not trivial, and to the best of the authors’ knowledge, there are no resources available for this specific matter. We define mapping/accumulation workflow as the selection of a given algorithm, its parameters, directionality, resampling strategy, and the definition of the minimum set of metrics and/or procedures for QA. Prior to defining a workflow, we propose to clearly define 1) what is the intended use of the DMA, and 2) which is the region of interest (ROI), which will then inform the expected anatomical variation. With these aspects, it is easier to identify the potential impact of dose uncertainty, such as illustrated in our DMAL, Fig. 4. Particularly, consider defining how to handle extreme anatomical variations (such as missing organs in the re-irradiation settings, or completely different anatomy as in inter-patient registrations).

Next, it is needed to assess whether DIR has fulfilled its task. Ideally, DIR would have aligned all the corresponding anatomy between the images, properly accounting for missing tissue (or organs). This could be translated to level 0 in the registration uncertainty assessment levels proposed in the TG-132 [1]. However, current registration algorithms often performed less optimal than this, with a recent publication comparing commercial systems reporting mean target registration error (TRE) ranging between 2.8 and 6.8 mm [16]. For DMA, locally aligned DIR, focused on the ROI (and/or the regions containing dose) would suffice (level 1, TG-132 [1]). However, these levels rely on quantifying the alignment of the anatomy, assumed to be defined on both images, using metrics such as TRE, mean distance to agreement, dice similarity coefficient (DSC). This presents a challenge as these anatomical landmarks/contours are often not available for one or both images. The validity of these geometrical metrics is limited to their direct vicinity, and cannot account for distortions far from their location [62]. DVF-based metrics (Jacobian determinant, inverse consistency error (ICE) and transitivity error (TE)) provide information in all locations where the DVF is defined. However, they don’t assess registration correctness but local volume changes (Jacobian) and consistency (ICE and TE), therefore they are insufficient on their own for QA [40]. We strongly recommend visual inspection of the deformed image and the transformation (deformed grid/DVF visualisation), focused on the ROI, as a strict minimum to provide a global assessment of DIR performance.

Testing the selected algorithm and parameters to phantoms (either physical or digital) also provides insight, as done at commissioning in clinical applications. Several phantoms have been developed and made available for the community. These include known ground truth features, such as landmarks, DVF and dose distribution [1]. For digital phantoms, a current limitation is that they are often provided in file formats other than DICOM, and treatment planning systems are relatively closed to import data beyond DICOM. Converting between file formats requires tools and custom scripts that may not be available on clinical systems, increases workload and potential for making mistakes, and hinders clinical adoption.

Next comes QA dose mapping itself. Where corresponding anatomical landmarks are visible in each image, the dose to the landmark can be sampled from both the original and mapped dose distributions. With accurate registrations, these doses should be equal. Assessing the discrepancies between their TRE and dose deviations may give an indication of the cause of the uncertainty, whether due to spatial registration inaccuracy or a second-order effect. The confidence in the landmark identification is a limitation. Furthermore, this assessment is valid in the landmarks’ proximity.

Other strategies were proposed to assess the impact of inverse (in)consistent registrations, for instance the DVH overlap method [63]. Using the terminology introduced in Fig. 1, two transformations are used: source-to-destination and its inverse, destinationto-source. Structures defined on the destination grid are propagated onto the source dose distribution, and the source dose distribution is mapped to the destination grid. The impact of inconsistencies in the registration within the structures is assessed by comparing the derived dose volume histograms (DVH) of dose/-contour sets. If the volume of the structure is conserved, the mapped DVH should be equal to the original DVH and imply minimal inverse consistency error (ICE). For this method, structures must be present in the destination image and the quantification would only be valid for the region delineated. Moreover, variations between original and mapped DVHs will depend on the structure volume; with small mapping errors exacerbated in small volume structures, while larger mapping errors may be masked in large volume structures. Another strategy used the inconsistencies of registration defined as the net displacement of every voxel in different structures after successive application of the forward and backward transformations, and summarised differences using DVH bands [64]. However, these methods in isolation do not provide enough information for QA.

Methods were proposed to quantify dose mapping uncertainty in a local scale, by generating uncertainty maps [41,42,65]. In general, these maps contain the standard deviations of different mapped doses, calculated on each voxel. Salguero and colleagues [41] proposed a general framework to estimate voxel-wise dose mapping uncertainty: First, a cluster of points is obtained for each voxel from an iterative DIR, where each iteration included an “artificial” perturbation in the registration. Next, the dispersion of these points is used to compute spatial uncertainty. Last, the spatial uncertainty estimates are used in combination with the mapped dose distribution to compute the point-by-point dose standard deviation. Another strategy relies on a deformation model created using principal component analysis to sample spatially-correlated uncertainties and quantify their impact in daily dose mapping [42]. Hub and colleagues [65] estimated dose mapping uncertainties based on varying b-spline coefficients. Dose mapping uncertainties maps allow to keep spatial components of uncertain regions, which can help decide whether the mapped dose is of use. Therefore, we recommend quantifying the impact of registration uncertainties on dose mapping after a global assessment of DIR performance using one of these strategies [41,42,65]. In this context, a clear challenge is the lack of software tools facilitating the quantification and visualisation of these uncertainty maps in clinical practice.

Finally, QA carried out with developer methods and tools cannot replace the assessment of the user. Therefore, verification of DIR and DMA results by the user is essential.

#### Radiobiological issues

An important point raised during workshop discussions is that dose is at the end of the day a surrogate for what we really want to quantify, that is the radiobiological effect of the treatment. As outlined by Jaffray and colleagues [66] in their QUANTEC vision paper, the delivered dose has been poorly understood. Recent advances in DIR are allowing the delivered dose to be more accurately defined. Now that the delivered physical dose can be investigated, how do we incorporate this information into our understanding of radiobiology? A good example of a study linking radiobiological endpoints to the delivered - not planned dose - was performed by Bohoudi and colleagues [35]. They sought to identify delivered dose parameters linked with bladder toxicity. Dose was accumulated for 101 prostate SBRT patients treated with ART. The accumulated bladder V20–32 Gy showed better correlation than the planned V20–32 Gy with an increase in International Prostate Symptom Score. However, there were large bladder volume variations, potentially impacting the accuracy of the DIR.

Estimating biological accumulated dose is not straight forward. The Linear quadratic model (LQM) assumes fractional doses of equal magnitude used in a power-to-n law. Given the dose, especially to OARs, can vary each treatment, it is not valid to accumulate linearly using DIR. The concept of total biological dose, bEQD_d_, has been introduced by Niebuhr et al [67] to address this issue. Briefly, the bEQD_d_, represents the total treatment dose that yields a given biological effect but takes each dose per fraction into account rather than an average. Niebuhr et al report that the bEQD_d_ was systematically higher than conventionally accumulated dose with differences in hot spots of 3.3–4.9 Gy for conventional and 8.4 Gy for hypofractionated prostate cancer treatment plans. Determining the impact of these differences for outcome modelling and adaptive strategies is still unclear.

## Discussion

In this manuscript, we presented the current landscape of dose mapping/accumulation in RT, visually via the DMAL, Fig. 4. DMAL connects the expected anatomical variations and the impact of dose mapping uncertainties for patient safety and can be used in the future for analysis and safety considerations of new use cases. Unlike the magnitude of anatomical variations, metrics to quantify the impact of uncertainties on patient safety are lacking. Open discussions in a multi-disciplinary team are essential to define the position of any new use case. In our approach, we identified three main levels in the impact of uncertainties on patient safety, which are related to how the mapped/accumulated dose is used in the use cases. For use cases in the right side of the DMAL, a full quantification of dose mapping uncertainties (including both correctness and consistency) is required. However, tools to quantify these are not available in commercial packages. Therefore, we recommend extreme caution, with a very limited use in clinical practice. Vendors are encouraged to develop and implement tools to streamline this quantification soon, and ideally incorporate these uncertainties in treatment plan optimisation.

We also presented considerations on the current challenges in DMA, going beyond DIR uncertainties, which is the main focus of published literature [2,15]. We argue that the discussion on energy/mass transfer vs direct dose mapping is irrelevant, since, if done correctly, the results should not differ. This is at the end of the day, an implementation choice. We recommend using DDM in DMA implementations. However, we acknowledge that this decision is not in the hands of most users. Thus, it is further recommended transparent communication from the vendors on the selected strategy implemented in their clinical software to raise awareness of the limitation and possible impact of these for the users.

To get a comprehensive understanding of a DMA system, we recommend following four QA steps: ensuring the appropriate mapping/accumulation workflow was followed, QA of the DIR result, QA of the DMA result, and finally a review of the impact the DMA uncertainties will have on the clinical application. Implementing a DMA workflow involves many inter-connected tasks and decisions, and requires a well-coordinated team including dosimetrists, physicists, and radiation oncologists each playing different roles to ensure patient safety. We discussed the limited use and availability of digital phantoms for clinical systems commissioning, especially as non-standard file formats hinder their use clinically. Moreover, specific phantoms to enable commissioning, and automatic identification of corresponding landmarks would be of use for dose mapping QA.

The last major issue discussed in our manuscript was the impact of radiobiological uncertainties in dose accumulation. Dose, as a surrogate of the radiobiological effect of the treatment, is poorly understood. However, with DMA tools, we can start gaining insights on the true correlation between delivered dose and treatment outcomes. Furthermore, converting to radiobiologically-corrected doses (EQD2 or BED) relies on the use of the LQM, which can be an oversimplification of the real biological effect of dose in different tissues. Even with their shortcomings, we recommend using biologically corrected doses when accumulating doses.

Advanced registration techniques including diffeomorphic, symmetric, and inverse consistent algorithms, have been available in research software for years [51], and continue to be further developed [68], but few commercial packages include these features, limiting their use in clinical practice. There are also still several open research questions, such as how to best account for (dis) appearing tissue and sliding motion, process longitudinal data, and utilise recent advances in learning-based approaches [68]. The challenge of producing better registrations for DMA requires understanding exactly how the registration will be used to map/accumulate dose, and how the results will be interpreted and utilised in the clinical workflow. Multi-disciplinary work, where physicists, radiobiologist, clinicians, and computer scientists/engineers collaborate closely together is key to make real progress with dose mapping addressing the issues raised in this paper and beyond.

Several aspects cannot be improved by algorithms. As mentioned before, dose mapping relies on the registration of the underlying anatomy captured in medical images. Therefore, dose mapping applications will be hindered by any limitations in image acquisition and reconstruction. Additionally, complex anatomical changes such as inelastic tumour regression happening during treatment [6,69] will completely mislead any intensity-based registration and may result in highly uncertain dose mapping. Even though methods were proposed to identify these regression modes [6], these have not yet been actively incorporated in DMA. Mitigation strategies are required in the meantime; again, quantification of dose mapping uncertainties is a must.

Our last take-home message is to strive for a context-driven DIR rather than a perfect DIR. This may mean sacrificing global registration accuracy to favour locally accurate registrations or have multiple registrations depending on the organ/application. Here, it is important to be aware of the limitations of the implemented DIR algorithm, which is essential to understanding the impact of DIR and dose mapping uncertainties in the context of clinical decisions and judgements. For some use cases, keep in mind that dose mapping (with properly quantified uncertainties) is a better alternative than no mapping.

### Recommendations for vendors

- Develop and implement tools and visualisation means, which can be run quickly and easily after every registration in the system, aiming at:
  - Analysing the resulting transformations. Whenever contour/-landmarks are available (or created via automatic segmentation), we recommend including distance metrics, as recommended by TG-132, but warning the user on their limited validity. We also recommend visualising the DVF, as well as generating the map of the determinant of the Jacobian (to highlight contractions, expansions and registration folds). Additionally, visualisation of the inverse-consistency metric, bending energy, harmonic energy, curl would be desirable.
  - Identifying dose gradients to highlight the regions of high dosemapping uncertainty. We recommend overlaying this on the images as well as on the DVF/Jacobian maps.
  - Applying multiple algorithms (or parameters for the same algo-rithm) to provide a range of plausible registrations and estimate dose mapping uncertainty from these. Visualization can be done as confidence bands around individual DVHs or uncertainty maps (such as proposed in [41,42,65]. This is key to enabling QA.
- Integrate tools for performing and evaluating dose mapping with other clinical software such as auto-contouring and treatment planning systems to provide a seamless and automated clinical workflow for adaptive radiotherapy.
- Allow the user to export registration results (including initial rigid/affine and subsequent DVFs) for external evaluation and/or comparisons. This would ideally be in a standardised, well-documented, and easy to read format. For the DVF, we recommend using standard file formats, such as the DICOM extension for Deformable Registration in Radiation Oncology (DRRO) proposed by the IHE Radiation Oncology Technical Framework [70].
- Generate clear descriptions of the workflow applied, to allow traceability for mapped doses.
- Include in all training/tutorials resources clear indications of the limitations of the registration algorithm (e.g., inability of registering disappearing tissues) and dose mapping strategy implemented (e.g., direction of registration and resampling strategy) and their impact in a variety of cases, including ‘simple’ and challenging cases, such that users can easily identify possible shortcomings during normal operation.
- Allow the user to specify regions of interest where the registration should be as accurate as possible.
- Implement built-in workflows for resampling dose following the advice in this paper.
- Benchmark tools and algorithms on public data sets and publish results.
- Implement state-of-the-art DIR algorithms into commercial products, especially if their aim is dose mapping/accumulation. Examples include diffeomorphic, inverse consistent, symmetric and sliding motion.

### Recommendations for users

- Clearly identify the purpose of the DMA, and relate it to the DMAL, to determine the potential impact of the dose mapping uncertainties. This should be done both, at commissioning and at QA time.
- Consider local dose mapping uncertainties, ideally quantified with tools provided by vendors, and the impact this uncertainty will have on the specific application. Getting the appropriate registration for the application/organ/region should be the first priority, which may mean that you sacrifice global registration accuracy, or have multiple registrations. Always, consider which alternative is better for the patient: uncertain dose mapping or not mapping the dose at all (e.g., using DVH statistics) in the context of extra workload.
- Be aware of the software limitations and how to assess uncertainties, both for DIR and for dose mapping. For this we recommend training to develop a clear and comprehensive understanding on:
  - The DIR software used in their practice, including limitations, and implementation decisions,
  - The evaluation of DIR, both qualitatively and quantitatively, [1]
  - The dose mapping workflow used in their clinical software, including highlighting the regions of high impact due to uncertainties such as regions of high dose gradient
  - The evaluation of DMA uncertainties, ideally with tools provided by vendors,
  - The proper use of tools provided by vendors for QA for DIR andDMA, and their importance in daily practice.
- If feasible, apply multiple algorithms (or multiple feasible parameters) for the same task to provide a range of plausible registrations, from which a dose mapping uncertainty measure can be derived for every registration run which can have direct patient impact.
- Document clearly the registration workflow followed to map/accumulate dose distributions for each patient and the QA results.
- Develop departmental procedures and policies to perform DMA consistently, ideally following international recommendations/guidelines. Important aspects to account for include achievable and required accuracies for use cases in different anatomical sites (keeping patient safety in mind), alternative approaches when the registration is not successful or the dose mapping is highly uncertain, and how to handle tissue not present in both images.

## Supplementary Material

Suppl
