# Supplementary material for: Applicability and usage of dose mapping/accumulation in radiotherapy
Source: Radiother Oncol. Author manuscript; Available in PMC 2025 Mar 4. (PMC11877414; doi:10.1016/j.radonc.2023.109527)
Supplement: Suppl [file NIHMS2052252-supplement-Suppl.docx]

# Supplementary materials

These materials supplement the manuscript entitled: **Applicability and usage of dose mapping/accumulation (DMA) in radiotherapy**. Note that references between this and the main document are not linked.

## Supplementary 1: Selected use cases

Three use cases from the DMAL were selected to be discussed in detail, highlighting critical issues, current limitations and safety concerns when using dose mapping/accumulation. Notice that these cases are in the extremes of DMAL, Figure 4 in the main manuscript.

#### Adaptive radiotherapy (ART)

ART enables adapting a treatment to variations on the patient anatomy, by using treatment images such as CT, CBCT, and MR [1]. The adaptation can be achieved online (same day) or offline (scheduled or ad hoc) [2].

Ideally, dose accumulation in ART would allow to optimise treatment fractions based on dose delivered in previous fractions, while properly accounting for tumour regression (that is, elastic and inelastic regression modes). This use can be translated to ‘incorporate’ in our DMAL uncertainty axis. However, due to several limitations in DIR algorithms, including the proper management of tumour regression, dose accumulation is mostly used to quantify the delivered dose to the tumour and critical organs when each treatment fraction is a different plan and inform replanning. Therefore, dose accumulation is currently not standard for offline/online ART.

Different approaches can be taken when dose is mapped and accumulated in the ART scenario. For example, one can map the dose in a ‘cascade style’, where the previous fraction is mapped to the current fraction [3] (Figure S1.1, top), or one can map all fractions to a single reference image (1-n registrations, a.k.a. back-propagation, etc), e.g., planning CT or the first fraction image (Figure S1.1, bottom). Depending on the used approach, registration uncertainties will impact differently: in the cascade approach, errors in early registrations are propagated, while in the 1-n approach progressive changes will result in more challenging registration for images acquired reaching the end of treatment. Moreover, the order in which the doses are mapped can change the accumulated dose result unless the algorithm is inverse-consistent and transitive [4].

In the DMAL, ART is positioned in the anatomical variation axis from small to intermediate changes (Figure 4). We note that for some sites sudden large changes can happen such as lung deflation; however, the treatment may be interrupted for a complete re-plan. Particular challenges under this context include (dis)appearing of tissue (e.g. tumour), large volume changes (e.g. bladder or intestines) and deformations as well as small volume for registration (e.g. penile bulb) and image field of view cropped images and dose warping (either via DDM or EMT). At the impact of dose mapping uncertainties axis, offline ART is positioned at the beginning of high as it is used informative (retrospective, offline) in a patient-specific setting which typically does not affect the patient/treatment directly. However, notice that if a new plan is created mid-way in treatment due to the patient’s anatomy changing dramatically, for example lung atelectasis, any uncertainty when estimating the previously given dose would systematically impact the new treatment, increasing the risk of impact of dose mapping on patient safety. Online ART on the other hand is positioned at high, whenever mapped/accumulated dose is directly used for treatment optimisation (such as in ‘incorporate’), as it may directly affect treatment outcome if mapping uncertainties result in cancerous regions being underdosed or OARs overdosed.

Caution is advised with plans adapted on accumulated doses. We currently recommend against daily plan adaptation based on dose accumulation in clinical settings when the mapped dose is directly incorporated in treatment optimisation. In particular, when soft-tissue contrast is limited, registration uncertainties will directly impact dose mapping [5]. Studies reporting whether accumulated dose helps to better estimate risks of toxicities are a way to demonstrate the value of the tool, for example, retrospective MRgRT dose accumulation study of Bohoudi et al. showed potential to reduce the risk of acute urinary toxicity using dose accumulation [2]. In this case, mapped and accumulated doses were used to inform treatments.


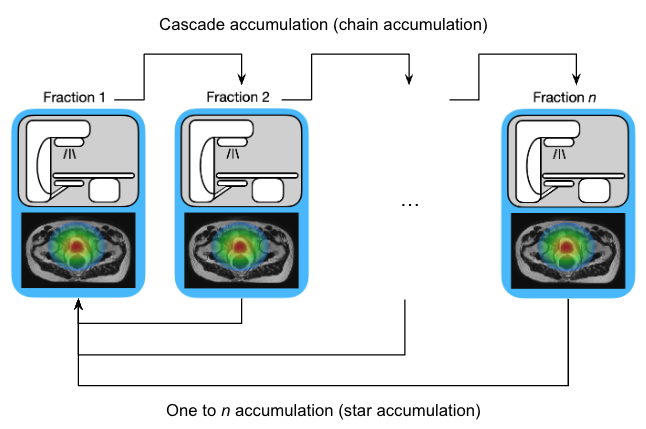


Figure S1.1: Illustration of the dose accumulation scenarios in ART. The ‘cascade’ accumulation (chain accumulation) is illustrated on the top. On the button, the ‘one to *n’* accumulation also known as star accumulation is illustrated.

#### Re-irradiation

In a recent consensus, re-irradiation has been defined as “a new course of radiotherapy, either to a previously irradiated volume (irrespective of concerns for toxicity) or where the cumulative dose raises concerns of toxicity [6]”, where often months or years happen between the two radiation treatments. Thus the key consideration in this scenario is the dose to critical organs that may have been previously irradiated. Re-irradiation is becoming more prevalent due to the ability of contemporary radiation therapy planning, delivery and image guidance technology to sculpt radiation dose distributions, minimising dose to previously treated critical organs/structures. The efficacy and safety of re-irradiation to tumour sites throughout the body has been demonstrated [7–15], however there still remains instances of high risk of catastrophic side effects due to excessive cumulative doses to critical organs/structures [16–20]. The risk of serious side effects is typically linked to complications that arise from maximum doses to structures (‘serial’ complications [21]). Notably, the cumulative maximum dose to a given organ/structure does not necessarily arise at the location of maximum dose from individual treatment courses. The concerns of toxicity from cumulative doses and whether the new course of RT overlaps with ir-radiated volume of previous course are both to be considered [6]. A lesser, but still important consideration is the less severe side effects which may impact patient quality of life such as brain necrosis and lung pneumonitis [16, 17, 22] are dependent not only on maximum organ doses but often volumetric metrics such as mean dose.

In re-irradiation, it is recommended that if the previous dose distributions are available, these are overlaid onto the new treatment planning image [6,23–26]. Dose from previous treatment(s) can be deformably mapped to a new planning image to assess delivered dose from previous treatment(s) to each spatial sub-volume of the organs/structures of interest as they appear in the new image.

The complexity of cumulative dose assessment depends on the quality of prior dose information, ranging from conservatively approximations of dose from previous treatment records in text or portal images, to dose accumulation of biologically equi-effective 3D dose [6]. From this information, the treating team can determine what dose can be safely delivered to each location of the critical organs/structures to minimise the risk of serious side effects from re-irradiation. It should be noted the importance of conversion of doses to biologically equieffective dose such as EQD2 via the linear quadratic formalism [24,26, 27]. Interestingly, there is no consensus on the adequate order for dose mapping and radiological correction, with approaches using both orders in the literature (e.g., map then correct [23] vs correct then map [28]). Additionally, tissue recovery factors, applied to the dose in specific organs typically spinal cord and central nervous system structures may or may not be included.

The position of re-irradiation in the DMAL is highly variable depending on the organ and dose to consider. Given the critical safety issue when quantifying the previously delivered dose (and cumulative dose from all courses), it is important that a high level of caution is exercised when applying deformable dose accumulation in re-irradiation. The position, size and shape of critical organs such as the spinal cord may be relatively constant over time-spans of months to years between treatment courses. As such, the anatomical variation is relatively small and rigid registration can be done with a relatively high degree of accuracy. Contrast this with tubular structures such as the bowel, where the filling and position will vary substantially not only between treatment courses but between each treatment session in a given treatment course. In this scenario, measurement of registration accuracy is extremely challenging and deformable dose mapping should be avoided; it is highly unlikely the accuracy of mapped dose can be determined in the instance where no clear corresponding anatomical landmarks exist. In between these two are deformable organs such as blood vessels and the oesophagus, where DIR accuracy can be improved and or assessed using contours in each image. In these cases deformable dose mapping may be considered.

Where mean doses are of importance, the variation in mean dose with different image registrations may be minimal, but likely depending on the volume of the organ; deformed mean dose to larger volume organs such as the lung and liver being more robust to uncertainties in dose mapping.

There is a high impact on patient safety from dose mapping uncertainty in organ maximum doses. To classify where high accuracy deformable dose mapping is required, one can perform a conservative estimate; in the absence of registration between the two courses, does the summation of the point maximum doses for a given organ from each individual course approach or exceed the tolerance for that organ? This is the worst case scenario; in this case, the application of deformable dose mapping should be accompanied with a high level of rigour to maximise the accuracy of the underlying deformation, and to quantify and communicate the uncertainty. The uncertainty may be estimated via performing the assessment using multiple methods such as the conservative approach described above, and by performing rigid registration in a localised region where high doses overlap.

A further consideration is the relevance of the planned dose in the absence of real-time dose accumulation during the course of each treatment using daily volumetric imaging. The estimated dose from the treatment planning scan to relatively rigid structures such as bones and spinal cord may be very close to the delivered dose. The planned dose to organs subject to a high level of random variation day-to-day however may be a poor estimate of the actual delivered dose to the organ. Thus using the planned dose grid from the original treatment course for highly variable structures may place a ceiling on the achievable accuracy of dose accumulation in the re-irradiation scenario.

Finally, we would recommend assessing the impact of registration uncertainties on the mapped dose. Methods have been proposed, see section first-order effects in the manuscript. As these tools are not currently available in treatment planning systems, performing different registrations (by varying either algorithm parameters/initial global alignment) and comparing the doses will help identify regions where the mapped dose is (un)stable.

#### Response assessment

Dose-response assessment tries to establish a relationship between the dose received by an organ (or subvolume of an organ) and clinical outcome. To increase the therapeutic ratio of RT, relations of true accumulated dose delivered to tumours and organs at risk (OARs), rather than the planned pretreatment dose, should be related to clinical outcome [29].

##### Per-patient dose-response assessment

The dose delivered during a treatment can be accumulated between treatment fractions to account for inter-fractional anatomical variations. Bohoudi et al. [3] used such an approach to estimate the dose delivered to the bladder for 101 prostate cancer patients treated with five fractions of magnetic resonance-guided stereotactic body radiation therapy. They showed that an increase in International Prostate Symptoms Score correlated better with bladder doses accumulated after three and five fractions of prostate RT than with pretreatment planned dose. This indicates that dose accumulation could improve understanding of dose-response relationships.

##### Voxel-wise analysis

Another implementation of dose mapping for dose response assessment is voxel-wise analysis. Voxel-wise analysis aims at exploring and locating dose-sensitive anatomical regions for a given treatment outcome. This analysis relies on mapping dose distributions of the studied cohort into a reference anatomy, commonly using DIR [30]. Voxel-wise analysis is one of the few inter-patient dose mapping cases in current use. As anatomical variations between patients are substantial, larger deviations are expected than the per-patient approach described above. For example, this methodology has been used to identify the association between dose to the obturatorial lymph node region and treatment failure after prostate RT [31,32], dose to the heart and overall survival after lung RT [33,34], dose to the masseter muscle and trismus after head and neck RT [35], among others.

Both approaches are used to quantify and compare dose distributions given to patients, in a retro-spective fashion. The results of these studies are hypothesis generating and form the basis of subsequent analysis aiming at confirming or rejecting the hypothesis. In this context, the impact of registration uncertainties is limited for a given patient.

## Supplement 2: List of discussed articles

Table S2.1. List of discussed articles on DIR and dose accumulation to identify current challenges during the online meetings and via email.

| **Ref.** | **Aim** | **Highlights, issues and challenges** | **Keywords** |
| --- | --- | --- | --- |
| Articles discussed during the online meetings | | | |
| Bosma et al., 2021  [36] | Influence of DIR algorithm, dose warping strategy, and signal-to-noise ratio on accumulated motion-compensated dose for MR-guided radiotherapy of prostate cancer. | The chosen dose warping strategy, DDM vs. EMT, affected the accumulated dose more than the DIR algorithm employed or the signal-to-noise ratio of the images. | DDM/EMT  motion-compensated dose accumulation (MCDA)  Prostate |
| Li et al., 2014  [37] | Comparison of dosimetric difference between DDM and EMT for 4D dose accumulation in lung SBRT. | Differences between DDM and EMT of 11% and 4% were observed for PTV and ITV minimum dose, respectively. | DDM/EMT |
| Rosu et al., 2005  [38] | Investigation of dose grid size and dose interpolation method on dose accumulation in a deforming lung anatomy. | The interpolation method alone did not yield clinically significant changes in dose accumulation results. These effects were larger in high dose gradient regions for larger dose grid sizes. | Dose grid size effects  (Lung) |
| Niebuhr et al., 2021  [39] | Based on daily CT images an assessment of biological dose accumulation was performed. | The usage of bEQDd ( total biological dose) in terms of dose accumulation, copes with a potential systematic inaccuracy predicting biological effects, ​​in particular, serial OAR in regions with dose gradients and for hypofractionation. | Biological & physical dose accumulation  Re-irradiation |
| Nix et al., 2021  [23] | Development of a software tool for for use with commercial treatment planning systems, taking radiation biology and anatomical changes into account, by comparing three dose summation approaches | Re-irradiation cases require fractionation correction to meaningfully assess cumulative doses and reduce the risk of unintentional OAR overdose. DIR can add clinically relevant information in selected cases, especially for significant anatomical change. | Re-irradiation  Pelvic  Dose summation  Radiobiologically |
| Kainz et al., 2022  [40] | Presentation of a new and practical QA method, the DVH overlap technic, for DIR-based dose accumulation | In order to assess DIR for dose accumulation, in which the ICE was low, the DVH overlay technique can be used. | QA  Inverse consistency |
| Paganelli et al., 2018  [41] | Enabling a patient-specific assessment of registration quality and subsequently improved personalised treatment. | Patient-specific evaluation of DIR with both geometric and dosimetric methods were reviewed; challenges in clinical use, guidelines, and validation of deformable dose propagation were discussed.. | QA  DIR  Patient specific validation |
| Palma et al., 2020  [30] | Review article on how to implement voxel-wise analysis to explore local anatomical radiosensitivity for a given outcome. | As DIR is often used to map the dose distributions of multiple patients to a common reference to analyse a given treatment outcome, several considerations are introduced in this article. In particular, different methods to evaluate inter-patient DIR are presented. | VBA  Image-based data-mining  Outcome modelling |
| Chetty and rosu-Bubulac, 2019  [42] | Review of currently available DIR and dose accumulation methods as well as their related clinical application. | Overview article of DIR used for dose accumulation, mainly focused on the registration aspects. | DIR  Methods of DA |
| García-Mollá et al., 2015  [43] | Validation of a CT-CBCT ART DIR algorithm by using POIs, and development of a method of encountering the results affecting the dose by using IC. | The algorithm is useful for head and neck patients, however clinical decisions for plan adaptation need to be ruled with respect to the variation of the results. | ART  Head and neck  Validation |
| Qin et al., 2018  [44] | Investigation of the differences of clinical-relevant doses in intensity-homogeneous organs warped by purely image-based and biomechanical model based DIR (BM-DIR). | For intensity-homogeneous organs BM-DIR could be useful, because these organs are affected with larger deformation, shrinkage. | Biomechanical model  Dose warming uncertainty |
| Roussakis et al., 2015  [45] | Workflow demonstration and evaluation for the interfractional deformable image registration followed by dose accumulation. | Under clinically realistic scenarios the dependability of a CT-to-CT commercial DIR-based dose warping and a workflow to assist clinical personnel to assess the results was shown. | QA, uncertainties  head and neck |
| Bohoudi et al. 2021  [3] | Identification of bladder dose parameters based on the accumulated treatment dose and investigation of the prediction of the accumulated treatment dose via the first three fraction doses. | In the prediction of urinary symptoms the accumulated dose was more specific than the planned dose, also the prediction of the accumulated dose via the first three fraction doses showed a positive correlation | Accumulated dose prediction,  Toxicity prediction |
| Ziegenhein et al. 2018  [46] | Development of a real-time dose reconstruction via energy/mass transfer mapping. | The developed method makes real-time dose reconstitution via energy/mass transfer. Clinically applicable with the caveat that more computing power is required. | EMT  Real-time dose reconstruction |
| Chao et al. 2012  [47] | Development of a retrospectively reconstruction method of the delivered dose by using DIR in relation to the anatomical changes. | The method provides verification of the treatment dose with potential use in ART. | Verification treatment dose  ART  Irradiation total body |
| Rigaud et al. 2019  [48] | Description of current main DIR applications, their methods and principles and the evaluation. | DIR should be evaluated with geometrical and dosimetric metrics appropriately. | DIR  QA |
| Additional discussed articles | | | |
| Yin et al., 2009  [49] | Introduces a new similarity measure, the sum of squared tissue volume difference (SSTVD), which accounts for density changes between CT images, thus ensuring mass is preserved by the registration | Over the registration pairs the SSTDV methods results showed a smaller average lendmark error than the SSD method. | Sum of squared tissue volume difference SSTVD |
| Frederick et al. 2022  [50] | Demonstration of a volumetric image-guided radiotherapy and dose accumulation approach to derive planning target volume (PTV) margins for a patient population. | The derived PTV margins were 3 and 5 mm and ensured that in 90% of the patients at 98% of the CTV  95% of the prescribed dose was delivered. | PTV margins  Oropharyngeal cancer  Breast cancer |
| Kim et al. 2021  [51] | Review for external beam radiation therapy and brachytherapy dose summation strategies | For clinical use DIR is still in development regarding accuracy and efficiency. From the effective biological dose a composted DVH of the biological dose summation (EBRT dose map and the IGBT fractions) can be generated but an accurate radiobiological parameter is tissue dependant. The limitations of these methods of approximating dose accumulation needs to be considered. | Dose summation  Biologic weighted dose maps |

## Bibliography

[1] S. Lim-Reinders, B. M. Keller, S. Al-Ward, A. Sahgal, and A. Kim, ‘Online Adaptive Radiation Therapy’, *Int. J. Radiat. Oncol. Biol. Phys.*, vol. 99, no. 4, pp. 994–1003, Nov. 2017, doi: 10.1016/j.ijrobp.2017.04.023.

[2] J. Bertholet *et al.*, ‘Patterns of practice for adaptive and real-time radiation therapy (POP-ART RT) part II: Offline and online plan adaption for interfractional changes’, *Radiother. Oncol.*, vol. 153, pp. 88–96, Dec. 2020, doi: 10.1016/j.radonc.2020.06.017.

[3] O. Bohoudi, A. M. E. Bruynzeel, S. Tetar, B. J. Slotman, M. A. Palacios, and F. J. Lagerwaard, ‘Dose accumulation for personalized stereotactic MR-guided adaptive radiation therapy in prostate cancer’, *Radiother. Oncol. J. Eur. Soc. Ther. Radiol. Oncol.*, vol. 157, pp. 197–202, Apr. 2021, doi: 10.1016/j.radonc.2021.01.022.

[4] E. T. Bender, N. Hardcastle, and W. A. Tomé, ‘On the dosimetric effect and reduction of inverse consistency and transitivity errors in deformable image registration for dose accumulation’, *Med. Phys.*, vol. 39, no. 1, pp. 272–280, Jan. 2012, doi: 10.1118/1.3666948.

[5] U. J. Yeo, J. R. Supple, M. L. Taylor, R. Smith, T. Kron, and R. D. Franich, ‘Performance of 12 DIR algorithms in low-contrast regions for mass and density conserving deformation’, *Med. Phys.*, vol. 40, no. 10, p. 101701, Oct. 2013, doi: 10.1118/1.4819945.

[6] N. Andratschke *et al.*, ‘European Society for Radiotherapy and Oncology and European Organisation for Research and Treatment of Cancer consensus on re-irradiation: definition, reporting, and clinical decision making’, *Lancet Oncol.*, vol. 23, no. 10, pp. e469–e478, Oct. 2022, doi: 10.1016/S1470-2045(22)00447-8.

[7] N. Riaz *et al.*, ‘A nomogram to predict loco-regional control after re-irradiation for head and neck cancer’, *Radiother. Oncol.*, vol. 111, no. 3, pp. 382–387, Jun. 2014, doi: 10.1016/j.radonc.2014.06.003.

[8] R. Rulach, G. G. Hanna, K. Franks, J. McAleese, and S. Harrow, ‘Re-irradiation for Locally Recurrent Lung Cancer: Evidence, Risks and Benefits’, *Clin. Oncol.*, vol. 30, no. 2, pp. 101–109, Feb. 2018, doi: 10.1016/j.clon.2017.11.003.

[9] A. J. Koong, D. A. S. Toesca, R. von Eyben, E. L. Pollom, and D. T. Chang, ‘Reirradiation with stereotactic body radiation therapy after prior conventional fractionation radiation for locally recurrent pancreatic adenocarcinoma’, *Adv. Radiat. Oncol.*, vol. 2, no. 1, pp. 27–36, Jan. 2017, doi: 10.1016/j.adro.2017.01.003.

[10] S. Myrehaug, H. Soliman, C. Tseng, C. Heyn, and A. Sahgal, ‘Re-irradiation of Vertebral Body Metastases: Treatment in the Radiosurgery Era’, *Clin. Oncol.*, vol. 30, no. 2, pp. 85–92, Feb. 2018, doi: 10.1016/j.clon.2017.11.005.

[11] L. J. Murray, J. Lilley, M. A. Hawkins, A. M. Henry, P. Dickinson, and D. Sebag-Montefiore, ‘Pelvic re-irradiation using stereotactic ablative radiotherapy (SABR): A systematic review’, *Radiother. Oncol.*, vol. 125, no. 2, pp. 213–222, Nov. 2017, doi: 10.1016/j.radonc.2017.09.030.

[12] M. Shanker, B. Chua, C. Bettington, M. C. Foote, and M. B. Pinkham, ‘Re-irradiation for recurrent high-grade gliomas: a systematic review and analysis of treatment technique with respect to survival and risk of radionecrosis’, *Neuro-Oncol. Pract.*, vol. 6, no. 2, pp. 144–155, Mar. 2019, doi: 10.1093/nop/npy019.

[13] M. G. Guren *et al.*, ‘Reirradiation of locally recurrent rectal cancer: A systematic review’, *Radiother. Oncol.*, vol. 113, no. 2, pp. 151–157, Nov. 2014, doi: 10.1016/j.radonc.2014.11.021.

[14] D. Owen *et al.*, ‘Challenges in Reirradiation of Intrahepatic Tumors’, *Semin. Radiat. Oncol.*, vol. 30, no. 3, pp. 242–252, Jul. 2020, doi: 10.1016/j.semradonc.2020.02.004.

[15] Y. S. Kim, ‘Reirradiation of head and neck cancer in the era of intensity-modulated radiotherapy: patient selection, practical aspects, and current evidence’, *Radiat. Oncol. J.*, vol. 35, no. 1, pp. 1–15, Mar. 2017, doi: 10.3857/roj.2017.00122.

[16] T. J. Kruser *et al.*, ‘Reirradiation for Locoregionally Recurrent Lung Cancer: Outcomes in Small Cell and Non–Small Cell Lung Carcinoma’, *Am. J. Clin. Oncol.*, vol. 37, no. 1, pp. 70–76, Feb. 2014, doi: 10.1097/COC.0b013e31826b9950.

[17] K.-L. Wu *et al.*, ‘Three-dimensional conformal radiotherapy for locoregionally recurrent lung carcinoma after external beam irradiation: A prospective phase I–II clinical trial’, *Int. J. Radiat. Oncol.*, vol. 57, no. 5, pp. 1345–1350, Dec. 2003, doi: 10.1016/S0360-3016(03)00768-5.

[18] B. J. Gebhardt *et al.*, ‘Carotid Dosimetry and the Risk of Carotid Blowout Syndrome After Reirradiation With Head and Neck Stereotactic Body Radiation Therapy’, *Int. J. Radiat. Oncol.*, vol. 101, no. 1, pp. 195–200, May 2018, doi: 10.1016/j.ijrobp.2017.11.045.

[19] M. Trovo *et al.*, ‘Stereotactic Body Radiation Therapy for Re-irradiation of Persistent or Recurrent Non-Small Cell Lung Cancer’, *Int. J. Radiat. Oncol.*, vol. 88, no. 5, pp. 1114–1119, Apr. 2014, doi: 10.1016/j.ijrobp.2014.01.012.

[20] M. W. McDonald, M. G. Moore, and P. A. S. Johnstone, ‘Risk of Carotid Blowout After Reirradiation of the Head and Neck: A Systematic Review’, *Int. J. Radiat. Oncol.*, vol. 82, no. 3, pp. 1083–1089, Mar. 2012, doi: 10.1016/j.ijrobp.2010.08.029.

[21] C. H. Crane, ‘Balancing Fractionation and Advanced Technology in Consideration of Reirradiation’, *Semin. Radiat. Oncol.*, vol. 30, no. 3, pp. 201–203, Jul. 2020, doi: 10.1016/j.semradonc.2020.02.009.

[22] C. Nieder, N. H. Andratschke, and A. L. Grosu, ‘Re-irradiation for Recurrent Primary Brain Tumors’, *Anticancer Res.*, vol. 36, no. 10, pp. 4985–4995, Oct. 2016.

[23] M. Nix *et al.*, ‘Dose summation and image registration strategies for radiobiologically and anatomically corrected dose accumulation in pelvic re-irradiation’, *Acta Oncol.*, vol. 61, no. 1, pp. 64–72, Jan. 2022, doi: 10.1080/0284186X.2021.1982145.

[24] K. C. Paradis *et al.*, ‘The Special Medical Physics Consult Process for Reirradiation Patients’, *Adv. Radiat. Oncol.*, vol. 4, no. 4, 2019, doi: 10.1016/j.adro.2019.05.007.

[25] E. Boman, M. Kapanen, L. Pickup, and S.-L. Lahtela, ‘Importance of deformable image registration and biological dose summation in planning of radiotherapy retreatments’, *Med. Dosim.*, vol. 42, no. 4, pp. 296–303, Dec. 2017, doi: 10.1016/j.meddos.2017.06.006.

[26] C. Schröder *et al.*, ‘Re-irradiation in the thorax – An analysis of efficacy and safety based on accumulated EQD2 doses’, *Radiother. Oncol.*, vol. 152, pp. 56–62, Nov. 2020, doi: 10.1016/j.radonc.2020.07.033.

[27] E. Boman, M. Kapanen, L. Pickup, and S.-L. Lahtela, ‘Importance of deformable image registration and biological dose summation in planning of radiotherapy retreatments’, *Med. Dosim.*, vol. 42, no. 4, pp. 296–303, Dec. 2017, doi: 10.1016/j.meddos.2017.06.006.

[28] E. D. Brooks *et al.*, ‘An algorithm for thoracic re-irradiation using biologically effective dose: a common language on how to treat in a “no-treat zone”’, *Radiat. Oncol.*, vol. 17, no. 1, p. 4, Jan. 2022, doi: 10.1186/s13014-021-01977-1.

[29] D. A. Jaffray, P. E. Lindsay, K. K. Brock, J. O. Deasy, and W. A. Tomé, ‘Accurate Accumulation of Dose for Improved Understanding of Radiation Effects in Normal Tissue’, *Int. J. Radiat. Oncol.*, vol. 76, no. 3, pp. S135–S139, Mar. 2010, doi: 10.1016/j.ijrobp.2009.06.093.

[30] G. Palma, S. Monti, and L. Cella, ‘Voxel-based analysis in radiation oncology: A methodological cookbook’, *Phys. Med.*, vol. 69, pp. 192–204, Jan. 2020, doi: 10.1016/j.ejmp.2019.12.013.

[31] M. G. Witte *et al.*, ‘Relating Dose Outside the Prostate With Freedom From Failure in the Dutch Trial 68 Gy vs. 78 Gy’, *Int. J. Radiat. Oncol.*, vol. 77, no. 1, pp. 131–138, May 2010, doi: 10.1016/j.ijrobp.2009.04.040.

[32] M. Witte, F. Pos, L. Incrocci, and W. Heemsbergen, ‘Association between incidental dose outside the prostate and tumor control after modern image-guided radiotherapy’, *Phys. Imaging Radiat. Oncol.*, vol. 17, pp. 25–31, Jan. 2021, doi: 10.1016/j.phro.2020.12.003.

[33] A. McWilliam, J. Kennedy, C. Hodgson, E. Vasquez Osorio, C. Faivre-Finn, and M. van Herk, ‘Radiation dose to heart base linked with poorer survival in lung cancer patients’, *Eur. J. Cancer*, vol. 85, pp. 106–113, Nov. 2017, doi: 10.1016/j.ejca.2017.07.053.

[34] L. Cella *et al.*, ‘Probing thoracic dose patterns associated to pericardial effusion and mortality in patients treated with photons and protons for locally advanced non-small-cell lung cancer’, *Radiother. Oncol.*, vol. 160, pp. 148–158, Jul. 2021, doi: 10.1016/j.radonc.2021.04.025.

[35] W. Beasley *et al.*, ‘Image-based Data Mining to Probe Dosimetric Correlates of Radiation-induced Trismus’, *Int. J. Radiat. Oncol.*, vol. 102, no. 4, pp. 1330–1338, Nov. 2018, doi: 10.1016/j.ijrobp.2018.05.054.

[36] L. S. Bosma, C. Zachiu, M. Ries, B. D. de Senneville, and B. W. Raaymakers, ‘Quantitative investigation of dose accumulation errors from intra-fraction motion in MRgRT for prostate cancer’, *Phys. Med. Biol.*, vol. 66, no. 6, p. 065002, Mar. 2021, doi: 10.1088/1361-6560/abe02a.

[37] H. S. Li *et al.*, ‘Direct dose mapping versus energy/mass transfer mapping for 4D dose accumulation: fundamental differences and dosimetric consequences’, *Phys. Med. Biol.*, vol. 59, no. 1, pp. 173–188, Jan. 2014, doi: 10.1088/0031-9155/59/1/173.

[38] M. Rosu, I. J. Chetty, J. M. Balter, M. L. Kessler, D. L. McShan, and R. K. Ten Haken, ‘Dose reconstruction in deforming lung anatomy: Dose grid size effects and clinical implications’, *Med. Phys.*, vol. 32, no. 8, pp. 2487–2495, 2005, doi: 10.1118/1.1949749.

[39] N. I. Niebuhr *et al.*, ‘Biologically consistent dose accumulation using daily patient imaging’, *Radiat. Oncol.*, vol. 16, no. 1, p. 65, Apr. 2021, doi: 10.1186/s13014-021-01789-3.

[40] K. Kainz *et al.*, ‘Use of a DVH overlay technique for quality assurance of deformable image registration-based dose accumulation’, *Med. Phys.*, vol. 49, no. 1, pp. 611–623, 2022, doi: 10.1002/mp.15375.

[41] C. Paganelli, G. Meschini, S. Molinelli, M. Riboldi, and G. Baroni, ‘Patient-specific validation of deformable image registration in radiation therapy: Overview and caveats’, *Med. Phys.*, vol. 45, no. 10, pp. e908–e922, Oct. 2018, doi: 10.1002/mp.13162.

[42] I. J. Chetty and M. Rosu-Bubulac, ‘Deformable Registration for Dose Accumulation’, *Semin. Radiat. Oncol.*, vol. 29, no. 3, pp. 198–208, Jul. 2019, doi: 10.1016/j.semradonc.2019.02.002.

[43] R. García-Mollá, N. de Marco-Blancas, J. Bonaque, L. Vidueira, J. López-Tarjuelo, and J. Perez-Calatayud, ‘Validation of a deformable image registration produced by a commercial treatment planning system in head and neck’, *Phys. Med.*, vol. 31, no. 3, pp. 219–223, May 2015, doi: 10.1016/j.ejmp.2015.01.007.

[44] A. Qin, J. Liang, X. Han, N. O’Connell, and D. Yan, ‘Technical Note: The impact of deformable image registration methods on dose warping’, *Med. Phys.*, vol. 45, no. 3, pp. 1287–1294, Mar. 2018, doi: 10.1002/mp.12741.

[45] Y. G. Roussakis, H. Dehghani, S. Green, and G. J. Webster, ‘Validation of a dose warping algorithm using clinically realistic scenarios’, *Br. J. Radiol.*, vol. 88, no. 1049, p. 20140691, May 2015, doi: 10.1259/bjr.20140691.

[46] P. Ziegenhein, C. Ph. Kamerling, M. F. Fast, and U. Oelfke, ‘Real-time energy/mass transfer mapping for online 4D dose reconstruction’, *Sci. Rep.*, vol. 8, p. 3662, Feb. 2018, doi: 10.1038/s41598-018-21966-x.

[47] M. Chao, J. Penagaricano, Y. Yan, E. G. Moros, P. Corry, and V. Ratanatharathorn, ‘Voxel-Based Dose Reconstruction for Total Body Irradiation With Helical TomoTherapy’, *Int. J. Radiat. Oncol. Biol. Phys.*, vol. 82, no. 5, pp. 1575–1583, Apr. 2012, doi: 10.1016/j.ijrobp.2011.01.021.

[48] B. Rigaud *et al.*, ‘Deformable image registration for radiation therapy: principle, methods, applications and evaluation’, *Acta Oncol.*, vol. 58, no. 9, pp. 1225–1237, Sep. 2019, doi: 10.1080/0284186X.2019.1620331.

[49] Y. Yin, E. A. Hoffman, and C.-L. Lin, ‘Mass preserving nonrigid registration of CT lung images using cubic B-spline’, *Med. Phys.*, vol. 36, no. 9Part1, pp. 4213–4222, 2009, doi: 10.1118/1.3193526.

[50] A. Frederick *et al.*, ‘An updated approach for deriving PTV margins using image guidance and deformable dose accumulation’, *Phys. Med. Ampmathsemicolon Biol.*, vol. 67, no. 7, p. 075004, Mar. 2022, doi: 10.1088/1361-6560/ac5ce5.

[51] H. Kim *et al.*, ‘Dose Summation Strategies for External Beam Radiation Therapy and Brachytherapy in Gynecologic Malignancy: A Review from the NRG Oncology and NCTN Medical Physics Subcommittees’, *Int. J. Radiat. Oncol. Biol. Phys.*, vol. 111, no. 4, pp. 999–1010, Nov. 2021, doi: 10.1016/j.ijrobp.2021.06.019.
